# Genomic and proteomic conversion of brain ischemia to Alzheimer’s disease

**DOI:** 10.3389/fcell.2026.1804251

**Published:** 2026-05-14

**Authors:** Ryszard Pluta, Marzena Ułamek-Kozioł

**Affiliations:** 1 Department of Pathophysiology, Medical University of Lublin, Lublin, Poland; 2 Department of Neurology, Institute of Psychiatry and Neurology, Warsaw, Poland

**Keywords:** Alzheimer’s disease, amyloid, amyloid precursor protein, apolipoproteins, apoptosis, autophagy, brain ischemia, genes

## Abstract

Despite many years of extensive research into the etiology and treatment of Alzheimer’s disease, based on the importance of amyloid and tau protein as causative factors, these studies have stalled, have not brought any breakthroughs and, most importantly, have not led to any final conclusions. Therefore, the ineffectiveness of the above-mentioned actions and the pressure from the community of people affected by Alzheimer’s disease forced the scientific community to change its way of thinking about the etiopathogenesis of this disease. This situation has prompted a group of scientists who have been studying the effects of brain ischemia for years to focus on post-ischemic changes, which–similarly to Alzheimer’s disease–predominate in the hippocampus, leading to the development of amyloid plaques, neurofibrillary tangles and ultimately to dementia. In this context, it has been proposed that brain ischemia may play an important role in driving amyloid and tau protein pathology in the development of Alzheimer’s disease. In this review, we present an update of extensive experimental and clinical studies conducted over several years on the role of brain ischemia in the neuropathogenesis of Alzheimer’s disease. Current advances in understanding the ischemic etiology of Alzheimer’s disease have revealed dysregulation of Alzheimer’s disease-associated genes, including secretases, amyloid precursor protein, apoptosis, autophagy, mitophagy, tau protein, α-synuclein, apolipoproteins, LRP1, and RAGE. This article presents the relationship between genes which dysregulation is a result of brain ischemia and the cellular and tissue neuropathology characteristic of Alzheimer’s disease and their proteins. These observations clearly indicate that, following brain ischemia, changes occur in the expression of Alzheimer’s disease-associated genes and in the folding of disease-associated proteins such as amyloid, tau protein, and α-synuclein. This leads to massive neuronal death and disruption of the neuronal network, ultimately leading to the development of Alzheimer’s disease-like dementia. Data indicate common genomic and proteomic factors in brain ischemia and Alzheimer’s disease. It seems that the brain ischemia model may be useful in determining the role of folding proteins and their genes dysregulation in Alzheimer’s disease. In the future, manipulation of genes and proteins associated with ischemia and ischemia-induced Alzheimer’s disease will likely provide new hope for developing causal therapies that are urgently needed to prevent or treat Alzheimer’s disease. The innovative/novel approach to the etiology of Alzheimer’s disease presented in this review will provide stakeholders with a glimpse into the future.

## 1 Introduction

On 3 November 1906, at a congress of German psychiatrists in Tübingen, Germany, Alois Alzheimer delivered a lecture entitled “A Peculiar, Severe Disease Process of the Cerebral Cortex” in a woman named Auguste Deter with symptoms of presenile dementia. He also presented the results of an autopsy of her brain, which revealed the presence of senile plaques and neurofibrillary tangles (Hippius and Neundörfer, 2003; Thakor et al., 2024). In 1910, the German psychiatrist Emil Kraepelin gave this disease the name “Alzheimer’s disease” (Yang et al., 2016; Ciurea et al., 2023). Currently, Alzheimer’s disease is the most common neurodegenerative disease, affecting approximately 50 million people worldwide. This number is estimated to increase to around 152 million by 2050 (Livingston et al., 2020; Xiaopeng et al., 2025). Alzheimer’s disease most commonly occurs in people aged 65 and older, with an average life expectancy of about 3–10 years (Liang et al., 2021). Depending on the stage of the disease, patients may experience cognitive and behavioral decline, initially manifesting as problems with memory and spatial orientation, as well as dysfunction in executive functions such as planning/organization, impaired reasoning and judgment, attention and concentration problems, personality and behavioral changes, mood changes, social withdrawal, agitation and aggression, paranoia/delusions, sleep disturbances, and ultimately dementia (Neupane and Hortobágyi, 2025). Currently, Alzheimer’s disease is the leading cause of dementia, affecting 60%–80% of the aging population worldwide, and the sixth leading cause of death (Garre-Olmo, 2018; Landeiro et al., 2018).

Alzheimer’s disease is a complex and multifactorial disease that affects all races and genders. Alzheimer’s disease is a common, progressive and chronic neurodegenerative disease of the brain, characterized by a long asymptomatic period, lasting up to 20 years (Elman-Shina and Efrati, 2022; Li X. Y. et al., 2023; Pluta et al., 2025a). The disease is characterized by progressive neurodegeneration of various brain structures at different times, in which characteristic neuropathological elements appear, such as amyloid plaques, neurofibrillary tangles, cerebral amyloid angiopathy, neuroinflammation, blood-brain barrier permeability, and brain atrophy.

Attempts to determine the cause of disease have been made for over a hundred years, but without much success. Over the years, many hypotheses have been proposed to understand the complex mechanisms underlying Alzheimer’s disease. These hypotheses are briefly discussed below. It should be emphasized that none of these theoretical and experimental proposals allowed for a full understanding of the disease or contributed to the development of an effective therapy modifying its course (Li X. Y. et al., 2023; Castellani et al., 2025; Hardy, 2025). It should be emphasized that despite decades of intensive research and enormous financial outlays, Alzheimer’s disease remains one of the most difficult neurological diseases to understand and treat. Interestingly, the key hypotheses discussed below are not mutually exclusive (Costa and Liloia, 2025). On the contrary, everything indicates that they interact and influence each other in a complex chain of events leading to the development of Alzheimer’s disease.

Alzheimer’s disease research is currently at a turning point, not so much because of a lack of data, but rather because of the formulation of hypotheses that assume they need to be confirmed, not disproved (Szabo, 2025). This crisis manifests itself in an overabundance of theory, a limited number of clearly comparable models, a proliferation of flexible predictions, and a shortage of truly differentiating experiments. This is due to institutional resistance to change and the policies of scientists themselves to maintain the dominant amyloid hypothesis (Szabo, 2025). In this scenario, the main goal is not to find a final theory, but to continuously formulate and multiply research questions. To solve the problem of Alzheimer’s disease, we need not only new theories but also knowledge that is more cautious in its claims and more rigorous in its approach. Research that is more open to the diversity of models and more rigorous in assessing their effectiveness. Only then will we be able to transform the current theoretical impasse into a real opportunity for progress. It seems, therefore, that the ischemic model of Alzheimer’s disease may offer such an opportunity, hope, and promise.

## 2 Classic hypotheses of the neuropathogenesis of Alzheimer’s disease

The amyloid hypothesis is still considered most important, pointing to a significant role of amyloid in the development of Alzheimer’s disease (Nasb et al., 2024; Chan, 2025; Costa and Liloia, 2025). For about 30 years, there has been a constant debate about the validity of this hypothesis, but without any significant conclusions (Li X. Y. et al., 2023; Castellani et al., 2025; Hardy, 2025). Objections to this hypothesis center on its apparent simplification and the fact that it is primarily based on familial, rare, early-onset Alzheimer’s disease and may not apply to the more common late-onset cases (Hardy, 2025). The amyloid hypothesis remains controversial because anti-amyloid therapies do not improve cognitive function in patients with Alzheimer’s disease, whereas cognitive impairment has not been observed in individuals with *postmortem* evidence of brain amyloidopathy (Kurkinen et al., 2023). This seemingly elegant, yet reductionist model has guided the development of Alzheimer’s disease treatments for years. However, the repeated failure of anti-amyloid therapies for Alzheimer’s disease to produce significant clinical effects has exposed the inadequacy of the amyloid theory as a unifying model explaining the cause(s) of the disease (Hein et al., 2026).

The concept of tau protein hyperphosphorylation highlights the role of neurofibrillary tangle formation in the development of Alzheimer’s disease (Nasb et al., 2024). It is important to note that the amyloid and tau protein theories are interconnected and mutually reinforcing, which indicates their weaknesses. Namely, the buildup of amyloid causes hyperphosphorylation of tau protein, which in turn leads to the development of neurofibrillary tangles and the death of neurons (Nasb et al., 2024; Zhuang et al., 2024). In turn, tau protein modifications increase the accumulation of amyloid and disrupts the mechanisms of its removal (Chan, 2025). Finally, the interaction of amyloid with tau protein causes a phenomenon that promotes neurodegeneration and cognitive impairment (Zhuang et al., 2024).

The cholinergic hypothesis posits that the cause of Alzheimer’s disease is the degeneration of cholinergic neurons, which leads to a decrease in the level of acetylcholine–a neurotransmitter associated with, among other things, learning and memory (Nasb et al., 2024; Costa and Liloia, 2025). It is believed that cholinergic deficiencies may be associated with amyloid accumulation and the development of neuroinflammation. It has been suggested that amyloid plaques may directly impair cholinergic neurotransmission, while neuroinflammation may additionally influence the loss of cholinergic neurons. On the other hand, cholinergic dysfunction may increase amyloid accumulation and promote neuroinflammation, thus triggering a vicious cycle of interactions (Chen Y. et al., 2022; Chen Z. R. et al., 2022).

Moreover, the neuroinflammation hypothesis suggests that chronic neuroinflammation plays a key role in the pathogenesis of Alzheimer’s disease (Costa and Liloia, 2025). There is a belief that neuroinflammation may be caused by amyloid plaques, but it may also trigger amyloid buildup and tau protein pathology. Moreover, inflammatory factors secreted by activated microglial cells promote amyloid formation, hinder its elimination and induce hyperphosphorylation of tau protein (Nasb et al., 2024). As a result, neuroinflammation further exacerbates neuroinflammatory responses, thus creating a self-perpetuating cycle that intensifies neuronal damage and disease progression (Miklossy, 2008).

The oxidative stress hypothesis proposes that oxidative changes contribute to the neuropathogenesis of Alzheimer’s disease (Nasb et al., 2024). Oxidative stress is believed to result from amyloid aggregation, which leads to the generation of reactive oxygen species and neuronal injury. In turn, oxidative stress may increase amyloid formation and tau protein hyperphosphorylation, which directly links this theory to the amyloid and tau protein hypotheses in the development of Alzheimer’s disease (Miranda et al., 2000). Furthermore, neuroinflammation can also exacerbate oxidative stress, contributing to additional neuronal damage and dysfunction (Singh et al., 2019).

The calcium signaling hypothesis is clearly related to other concepts regarding the causes of Alzheimer’s disease (Nasb et al., 2024). For example, the amyloid theory proposes that the formation of amyloid plaques in the brain causes disturbances in calcium homeostasis. This interaction suggests that abnormal calcium signaling may both contribute to and be a consequence of amyloid deposition (Abramov et al., 2004). Furthermore, abnormal calcium signaling may also contribute to excessive phosphorylation of tau protein, which leads to the formation of neurofibrillary tangles and, consequently, to neuronal dysfunction and death (Datta et al., 2021).

The mitochondrial theory suggests that defects in mitochondrial activity cause the occurrence and progression of Alzheimer’s disease (Nasb et al., 2024). Much evidence suggests that mitochondrial dysfunction is a key element in the development of Alzheimer’s disease, in conjunction with other hypotheses of disease (Wang et al., 2020). Mitochondrial dysfunction is thought to be linked to the buildup of amyloid and misfolded tau protein (Schmitt et al., 2012). Malfunctioning mitochondria produce reactive oxygen species and thus affect the metabolism of amyloid precursor protein, resulting in increased amyloid production (Bhat et al., 2015). In turn, amyloid disrupts the functioning of mitochondria, affecting their dynamics, oxidative phosphorylation and membrane potential, which leads to a vicious circle (Nasb et al., 2024). In addition, modified tau protein also affects mitochondrial transport, bioenergetics, dynamics and function (Perez et al., 2018). Then, impaired mitochondrial activity leads to a reduction in ATP production, which is necessary for synaptic transmission (Pluta et al., 2011). Interestingly, the neuronal cells of the cholinergic system are particularly sensitive to energy deficiency, which leads to cholinergic deficit (Perez et al., 2018). On the other hand, impaired cholinergic activity may also affect mitochondrial behavior by limiting the control of their function by acetylcholine (Chen Y. et al., 2022; Chen Z. R. et al., 2022). Furthermore, mitochondrial dysfunction can induce neuroinflammation and oxidative stress, and these phenomena in turn contribute to secondary mitochondrial dysfunction, leading to a vicious cycle that drives the neuropathology of Alzheimer’s disease (Bhat et al., 2015).

Mitochondrial and vascular disorders in Alzheimer’s disease have been shown to be closely linked. Limited blood flow to the brain disrupts mitochondrial activity, limiting the delivery of nutrients and oxygen to neuronal cells, leading to energy deficiencies and increased oxidative stress in mitochondria (Pluta et al., 2011). On the other hand, dysfunctional mitochondria release mediators that affect the functioning of brain blood vessels by disrupting endothelial cell function and blood-brain barrier permeability, further contributing to vascular damage in Alzheimer’s disease (Nasb et al., 2024).

While each hypothesis provides valuable information, none of them is sufficient in isolation to explain the full complexity of Alzheimer’s disease. In this review, by conceptualizing Alzheimer’s disease as a network disease, we highlight the need to move beyond isolated hypotheses and toward integrative models that can inform biomarker discovery, therapeutic development, and precision medicine approaches. For decades, Alzheimer’s disease research has been dominated by reductionist approaches that analyze individual hypotheses in isolation, such as amyloid deposition, tau protein pathology, or cholinergic deficiencies (Szabo, 2025). Although each of these hypotheses has led to valuable mechanistic insights, the continued failure of single-target therapies highlights the limitations of such isolated thinking. Now the emerging view is that Alzheimer’s disease is not a linear cascade, but rather a systemic disorder resulting from the interconnection of multiple dysregulated networks that normally maintain neuronal homeostasis (Hein et al., 2026).

All these hypotheses make sense, but they do not explain anything definitively. However, the ischemic hypothesis we propose considers antecedent factors such as impaired cerebral circulation, as well as downstream factors including neuronal loss, neuroinflammation, amyloid accumulation, tau protein dysfunction and etc. This article discusses the ischemic model of Alzheimer’s disease in detail and uses observed cases of cerebral ischemia and Alzheimer’s disease to explain this model, and *vice versa*.

## 3 Clinical observations suggesting a link between ischemia and Alzheimer’s disease

The ischemic hypothesis was developed based on epidemiological studies that showed a significant association between cerebral circulatory insufficiency, such as microinfarction, local, complete or silent ischemia and transient ischemic attacks, and the occurrence and development of cognitive impairment and dementia characteristic of Alzheimer’s disease (Ganesh and Barber, 2022; Ismail et al., 2022; Rost et al., 2022; Kamatham et al., 2024; Filler et al., 2025). Additional inspiration for the development of the ischemia puzzle was the discovery of platelets outside the cerebral vascular bed after experimental ischemia in electron microscopy studies (Pluta et al., 1994b). In this context, it is important to note that platelets contain large amounts of amyloid precursor protein and amyloid in the cytoplasm. It should be emphasized that systemic vascular risk factors, such as atherosclerosis, hypertension and diabetes, are factors causing cerebral ischemia with increased permeability of the blood-brain barrier, and at the same time risk factors for the development of Alzheimer’s disease (Tini et al., 2020). In patients after cerebral ischemia, amyloid deposition was found in the blood vessels of the meninges, in the cerebral cortex and subcortex, and in the hippocampus (Qi et al., 2007; Gemmell et al., 2012; Gemmell et al., 2014).

It has been shown that up to 92% of patients with Alzheimer’s disease have atherosclerotic changes in the brain, which are associated with cerebral amyloid angiopathy, loss of myelin in the white matter and progression of the disease (Tian et al., 2004). Additionally, single-photon emission computed tomography has shown that Alzheimer’s disease patients have reduced blood flow in different areas of the brain (Staff et al., 2000). It should be emphasized that an early symptom of Alzheimer’s disease is reduced blood flow in the brain (Korte et al., 2020; Goldsmith, 2022; Chan, 2025). These data add to the growing body of evidence that cerebral blood flow insufficiency in the form of ischemia significantly contributes to cognitive impairment and the development of Alzheimer’s disease-type dementia (Ganesh and Barber, 2022; Ismail et al., 2022; Rost et al., 2022; Kamatham et al., 2024; Filler et al., 2025). Reinforcing the above observations, increasing cerebral blood flow has been shown to improve cognitive function in advanced Alzheimer’s disease (Bracko et al., 2020). Moreover, it has been shown that in the brains of patients with Alzheimer’s disease, significantly more amyloid accumulates in the walls of cerebral blood vessels than in control people of the same age, which significantly affects the state of blood supply to the brain (Nasb et al., 2024).

### 3.1 Alzheimer’s disease-associated proteins in post-ischemic human brain and blood

Other studies of post-ischemic brains have revealed amyloid deposits: diffuse and senile plaques as in Alzheimer’s disease, diffuse, punctate deposits in the brain cortex associated with cerebral blood vessels, and amyloid accumulations located in the border zones of arteries in the cerebral cortex and ischemia-sensitive areas (Jendroska et al., 1995; Wisniewski and Maslinska, 1996; Jendroska et al., 1997; Frolov et al., 2025). Furthermore, clinical studies have shown elevated blood amyloid levels in cases of brain damage caused by ischemia (Lee et al., 2005; Zetterberg et al., 2011; Liu et al., 2015; Tarkowska et al., 2023). Increased tau protein levels have also been found in the blood serum of humans after brain ischemia, which is most likely related to the progressive damage of neurons after ischemia (Bitsch et al., 2002; Kurzepa et al., 2010; Bielewicz et al., 2011; Mörtberg et al., 2011; Randall et al., 2013; Lasek-Bal et al., 2016; De Vos et al., 2017; Tarkowska et al., 2023). Neurofibrillary tangles, characteristic of Alzheimer’s disease, have also been detected in the brains of patients after ischemia (Kato et al., 1988; Hatsuta et al., 2019; Frolov et al., 2025). In another study, it was found that the level of oligomeric form of α-synuclein in red blood cells of patients after brain ischemia was significantly higher than in healthy individuals (Zhao et al., 2016).

Furthermore, amyloid buildup has been shown to rise with age, which connects with the number of amyloid plaques (Frolov et al., 2025). Also, amyloid most often accumulates in the middle layers of the cerebral cortex, which are very susceptible to ischemia. Studies of brains after global ischemia revealed strong amyloid staining in neurons and perivascular spaces (Wisniewski and Maslinska, 1996). Neuronal staining was dependent on brain structure, but cortical neurons stained most intensely. In the hippocampus, neurons in the CA2 and CA3 areas stained more intensely than neurons in the CA1 region. In contrast, staining of dentate gyrus neuronal cells was clearly weak. Amyloid staining of neurons disappeared in areas of ischemic damage, whereas neurons adjacent to these regions showed strong staining for apolipoprotein E. It should be emphasized that some neuronal cells stained for apolipoprotein E were also stained with antibodies directed against tau protein. Ependymal and epithelial cells stained intensely for amyloid. Moreover, in the brains after ischemia, the presence of fibrillar amyloid plaques stained with thioflavin S and stained with the Bielschowsky method in the cerebral cortex was observed (Wisniewski and Maslinska, 1996; Frolov et al., 2025). However, the blood vessels of the gray and white matter were surrounded by amyloid deposits that took the shape of a cuff. In all brains examined post-ischemia, amyloid accumulation was found around the blood-brain barrier vessels. The accumulation of amyloid around the blood-brain barrier vessels indicates its very likely blood origin. Indirectly, increased blood amyloid levels after cerebral ischemia confirm the above suggestion (Lee et al., 2005; Zetterberg et al., 2011; Liu et al., 2015; Tarkowska et al., 2023).

According to another study, apolipoprotein E and β-amyloid peptide 1–40 and 1–42 were detected in the hippocampus after ischemia (Qi et al., 2007). Moreover, elevated blood amyloid levels correlated with poor clinical prognosis after ischemic brain injury (Zetterberg et al., 2011). The above evidence supports the hypothesis that brain ischemia may play a key role in the process of amyloidogenesis during the development of Alzheimer’s disease. Moreover, in patients after global cerebral ischemia, an increased level of tau protein in the blood was found, which is most likely related to the development of additional neuronal damage during recirculation (Mörtberg et al., 2011; Randall et al., 2013).

Interestingly, in the brains of patients after complete ischemia, staining of the receptor for advanced glycation end products (RAGE) was found in the epithelial cells of the choroid plexus and in the ependymal cells lining the lateral ventricles of the brain (Maślińska et al., 2011). These cells form both the blood-cerebrospinal fluid barrier and the cerebrospinal fluid-brain barrier. Furthermore, amyloid has been detected in the blood vessels of the choroid plexus and in the basement membrane of the choroid plexus epithelium (Maślińska et al., 2011). Amyloid was detected in numerous cytoplasmic vacuoles of the choroid plexus ependymal and epithelial cells, and the contents of these vacuoles gradually emptied. These observations indicate that choroid plexus epithelium and ependymal cells, which have a receptor for advanced glycation end products, play a significant role in amyloid deposition in the brain tissue but also provide a site from which amyloid can be cleared.

### 3.2 Dementia following brain ischemia in humans

A related consequence of the development of pathological changes in the brain after ischemia is the slow but progressive development of dementia (Gemmell et al., 2012; Gemmell et al., 2014; Brainin et al., 2015; Mok et al., 2016; Portegies et al., 2016; Kim and Lee, 2018; Frolov et al., 2025). Dementia is the worst consequence of ischemic brain damage in patients and accounts for approximately 20% of all diagnosed dementia cases (Fillit and Hill, 2002). Worldwide, the percentage of people with dementia after ischemic stroke ranges from 5% to 50% depending on diagnostic criteria, population demography, and geographic location (Surawan et al., 2017). In fact, there is now no doubt that post-ischemic dementia shares many common mechanisms with the late development of sporadic Alzheimer’s disease cases. There is a high probability that ischemic brain damage may precede the onset of Alzheimer’s disease-like dementia and cause all the consequences associated with the development of this type of dementia. Post-ischemic dementia associated with progressive, delayed secondary changes occurs in individuals suffering from transient ischemic attacks, lacunar, focal, total and silent ischemia in a progressive manner (Bornstein et al., 1996; Pinkston et al., 2009; Gemmell et al., 2012; Mok et al., 2016; Bivard et al., 2018). About 70% of patients 1 year after brain ischemia have mild to severe cognitive impairment (Rasquin et al., 2005; Ihle-Hansen et al., 2011; Pluta, 2024). Epidemiological studies have shown that the incidence of dementia in survivors of cerebral ischemia is about nine times higher than in the control group (Tatemichi et al., 1992; Pohjasvaara et al., 1998; Madureira et al., 2001; Surawan et al., 2017). Studies of patients at different times after brain ischemia have shown that the incidence of dementia was 7% within 1 year (Tatemichi et al., 1990), 10%–21% within 3 years (Henon et al., 2001), 22% within 4 years (Altieri et al., 2004), 15%–32% within 5 years (Bornstein et al., 1996) and 23% within 10 years of follow-up (Kokmen et al., 1996). However, after lacunar infarction it is 4–12 times more common than in healthy individuals (Loeb et al., 1992). In studies of individuals who experienced an episode of lacunar cerebral ischemia, dementia occurred in 5%–23% of patients after 1–4 years (Loeb et al., 1992; Samuelsson et al., 1996). Overall, after recurrent ischemic stroke, the incidence of dementia was 33% (Surawan et al., 2017).

## 4 Conceptual characteristics and details of the ischemic model of Alzheimer’s disease

Cerebral ischemia and Alzheimer’s disease initially present with divergent symptoms but ultimately converge toward common endpoints. Pathological overlap between the two diseases is mutual and is associated with progressive cognitive decline and disease progression over time. Ischemia induces amyloid and tau protein pathology, cholinergic dysfunction, neuroinflammation, oxidative stress, recurrent circulatory disturbances, and other changes, creating a vicious cycle (Humpel and Marksteiner, 2005; Nasb et al., 2024). The cumulative effect of these interactions is a network of interconnected neuropathological phenomena that contribute to the slow and prolonged progression of Alzheimer’s disease. The proposed ischemic model of Alzheimer’s disease reliably reflects the genotype and phenotype of disease-related events while highlighting its inherent advantages. The paradigm shift from amyloid as the primary cause of Alzheimer’s disease to ischemic factor allows for a comprehensive, step-by-step explanation of Alzheimer’s disease etiology, without the constraints of transgenic models. Below, we present updated data on the ischemic model of Alzheimer’s disease.

### 4.1 Changes in Alzheimer’s disease-related genes

Investigations revealed alterations in the expression of genes related to amyloid precursor protein metabolism in the CA1, CA3, and temporal and frontal cortex (Tables 1 and 2) (Kocki et al., 2015; Pluta et al., 2016a; Pluta et al., 2016b; Pluta et al., 2020; Czuczwar et al., 2024; Pluta et al., 2026b). Dysregulation of *β-secretase* (*BACE1*), *presenilin 1* and *2* (*PSEN 1* and *2*), and *amyloid precursor protein* (*APP*) in CA1 included all studied genes at 2-, 7-, and 30-day post-ischemia (Table 1). However, the changes in the expression of these genes and *α-secretase* (*ADAM10*) in the CA3 area of the hippocampus were less pronounced, did not affect amyloid production within 2–30 days after ischemia, and did not affect all genes (Table 1). Genes in this area were also evaluated after 1, 1.5 and 2 years of ischemia, where dysregulation was more severe but also without affecting amyloid production (Table 1). Gene dysregulation in the temporal cortex in the period 2–30 days post-ischemia was not always observed and did not concern all genes, and its intensity was much lower than in the CA3 area during the same period (Tables 1 and 2). However, in the frontal cortex after 2–30 days and 0.5–2 years post-ischemia, the changes in expression were more intense and differentiated than in the temporal cortex (Table 2).

**TABLE 1:** Expression of amyloid precursor protein processing genes in the hippocampus after brain ischemia in female rats, with survival from 2 days to 2 years (Kocki et al., 2015; Pluta et al., 2020; Czuczwar et al., 2024).

Survival genes	2 days	7 days	30 days	0.5 years	1 year	1.5 years	2 years
CA1 field							
*APP*	↑	↑	↑	ND	ND	ND	ND
*BACE1*	↑	↑	↓	ND	ND	ND	ND
*PSEN1*	↑	↑	↓	ND	ND	ND	ND
*PSEN2*	↑	↑	↓	ND	ND	ND	ND
CA3 field							
*APP*	↔	↑	↔	ND	↑	↑	↑
*ADAM10*	↓	↓	↓	ND	↑	↑	↑
*BACE1*	↓	↓	↑	ND	↑	↓	↑
*PSEN1*	↑	↑	↔	ND	↑	↔	↑
*PSEN2*	↔	↓	↑	ND	↑	↔	↑

Expression: ↑ increase; ↓ decrease; ↔ oscillation around control values; ND, no data. Genes: *APP*, amyloid precursor protein; *ADAM10*, α-secretase; *BACE1*, β-secretase; *PSEN1*, presenilin 1; *PSEN2*, presenilin 2.

**TABLE 2:** Expression of amyloid precursor protein processing genes in the temporal and frontal cortex after brain ischemia in female rats, with survival from 2 days to 2 years (Pluta et al., 2016a; Pluta et al., 2016b; Pluta et al., 2026b).

Survival genes	2 days	7 days	30 days	0.5 years	1 year	1.5 years	2 years
Temporal cortex							
*APP*	↓	↑	↑	ND	ND	ND	ND
*BACE1*	↑	↔	↔	ND	ND	ND	ND
*PSEN1*	↔	↔	↔	ND	ND	ND	ND
*PSEN2*	↑	↔	↔	ND	ND	ND	ND
Frontal cortex							
*APP*	↑	↓	↔	↓↓	↑↑	↑↑↑	↓↓
*ADAM10*	↑↑	↔	↔	↓	↑↑↑	↔	↔
*BACE1*	↔	↓	↓↓↓	↓↓	↑↑	↔	↔
*PSEN1*	↑	↔	↔	↓	↑↑	↔	↔
*PSEN2*	↑	↓	↔	↓	↑↑	↔	↔

Expression: ↑ increase; ↓ decrease; ↔ oscillation around control values; ND, not data. Genes: *APP,* amyloid precursor protein; *ADAM10,* α-secretase; *BACE1*, β-secretase; *PSEN1,* presenilin 1; *PSEN2,* presenilin 2.

Following cerebral ischemia, changes in the expression of *autophagy* (*BECN1*), *mitophagy* (*BNIP3*) and *apoptosis* (*CASP3*) were revealed, which are related with neuronal death (Table 3) (Ułamek-Kozioł et al., 2016; Ułamek-Kozioł et al., 2017; Ułamek-Kozioł et al., 2019; Pluta et al., 2024; Pluta et al., 2025a). *BECN1* activity in the CA1 area after ischemia and survival of 2–30 days oscillated around control values (Table 3). *BNIP3* overexpression in CA1 was revealed on day 2 post-ischemia, whereas on days 7–30 it was within the control range (Table 3). *CASP3* overexpression in the CA1 region was detected 2 days post-ischemia and was within the control range on days 7–30 (Table 3). Post-ischemic *BECN1* expression in the CA3 area fluctuated around control values at day 2, and 0.5, and 1.5 years, was decreased at day 7, and increased at day 30, and 1, and 2 years (Table 3). Post-ischemia, *BNIP3* expression was within control values at 2 and 30 days and after 0.5 and 1.5 years (Table 3). While on day 7 of survival there was a decrease in expression, on years 1 and 2 there was an increase. *CASP3* expression was at control values on days 2–30 and 1.5 years post-ischemia and increased at years 0.5, 1, and 2 (Table 3). *BECN1* overexpression was noted in the temporal cortex on day 2 post-ischemia. However, on days 7–30 it was within the control values (Table 4). On the second day of post-ischemia, *BNIP3* expression was decreased, on the seventh day it was significantly increased, and on the 30th day it was within the control values (Table 4). *CASP3* expression in the temporal cortex on days 2–30 following ischemia was within control values (Table 4). *BECN1* overexpression in the frontal cortex post-ischemia was observed on day 2 and 0.5, 1 and 2 years, and at the remaining times it oscillated around control values (Table 4). *BNIP3* expression on days 2–30 and after 1.5 years was within the control values, and was overexpressed after 0.5, 1 and 2 years. *CASP3* was overexpressed after ischemia at day 2 and 0.5, 1, and 2 years, and at other times it was close to control (Table 4).

**TABLE 3:** Autophagy, mitophagy, and apoptosis genes expression in the hippocampus after brain ischemia in female rats surviving from 2 days to 2 years (Ułamek-Kozioł et al., 2017; Ułamek-Kozioł et al., 2019; Pluta et al., 2024).

Survival genes	2 days	7 days	30 days	0.5 years	1 year	1.5 years	2 years
CA1 field							
*BECN1*	↔	↔	↔	ND	ND	ND	ND
*BNIP3*	↑	↔	↔	ND	ND	ND	ND
*CASP3*	↑↑↑	↔	↔	ND	ND	ND	ND
CA3 field							
*BECN1*	↔	↓	↑	↔	↑↑↑	↔	↑↑
*BNIP3*	↔	↓	↔	↔	↑↑↑	↔	↑
*CASP3*	↔	↔	↔	↑↑	↑↑↑	↔	↑↑

Expression: ↑ increase; ↓ decrease; ↔ fluctuation around control values; ND, no data. Genes: *BECN1*-autophagy; *BINP3*-mitophagy; *CASP3*-apoptosis.

**TABLE 4:** Autophagy, mitophagy, and apoptosis genes expression in the temporal and frontal cortex after brain ischemia in female rats surviving from 2 days to 2 years (Ułamek-Kozioł et al., 2016; Pluta et al., 2025a).

Survival genes	2 days	7 days	30 days	0.5 years	1 year	1.5 years	2 years
Temporal cortex							
*BECN1*	↑	↔	↔	ND	ND	ND	ND
*BNIP3*	↓↓	↑↑↑	↔	ND	ND	ND	ND
*CASP3*	↔	↔	↔	ND	ND	ND	ND
Frontal cortex							
*BECN1*	↑↑↑	↔	↔	↑	↑↑↑	↔	↑
*BNIP3*	↔	↔	↔	↑	↑↑↑	↔	↑
*CASP3*	↑↑	↔	↔	↑	↑↑↑	↔	↑

Expression: ↑ increase; ↓ decrease; ↔ fluctuation around control values; ND, no data. Genes: *BECN1*-autophagy; *BINP3*-mitophagy; *CASP3*-apoptosis.

Changes in the expression of *tau protein* (*MAPT*), *α-synuclein* (*SNCA*), low-density lipoprotein receptor-related protein 1 *LRP1* and *RAGE* were also examined following brain ischemia (Pluta et al., 2018a; Pluta et al., 2020; Pluta et al., 2023a; Czuczwar et al., 2024; Pluta et al., 2025c; Pluta et al., 2026b). In the CA1 region, *MAPT* expression was elevated on day 2 and fluctuated around control values 7–30 days after ischemia (Table 5). Overexpression in CA3 *MAPT* was revealed 7–30 days and 1–2 years after ischemia (Table 5). *SNCA* expression post-ischemia was decreased after 2 days and 2 years, oscillated around the control value at 0.5 years, and was overexpressed at other times (Table 5). *LRP1* expression was decreased from day 2 to 2 years following ischemia. *RAGE* expression was increased within 7–30 days after ischemia and decreased during the remaining observation periods (Table 5). In the frontal cortex after ischemia, *MAPT* expression oscillated around control values at day 30, was excessive at 1 and 1.5 years, and was below control values at the remaining times (Table 5). During 2 days and 1 and 1.5 years, *SNCA* expression was increased, and during 7 days and 2 years it was decreased, and in the remaining periods it remained within the control values (Table 5).

**TABLE 5:** Tau protein, α-synuclein, low-density lipoprotein receptor-related protein 1 and receptor for advanced glycation end products genes expression in the CA1 and CA3 areas of hippocampus and temporal cortex after brain ischemia in female rats surviving from 2 days to 2 years (Pluta et al., 2018a; Pluta et al., 2020; Pluta et al., 2023a; Czuczwar et al., 2024; Pluta et al., 2025c; Pluta et al., 2026b).

Survival genes	2 days	7 days	30 days	0.5 years	1 year	1.5 years	2 years
CA1 field							
*MAPT*	↑	↔	↔	ND	ND	ND	ND
CA3 field							
*MAPT*	↔	↑	↑	N.A.	↑	↑	↑
*SNCA*	↓	↑↑	↑	↔	↑↑↑	↑↑	↓↓
*LRP1*	↓	↓	↓	↓	↓	↓	↓
*RAGE*	↓	↑	↑	ND	↓	↓	↓
Frontal cortex							
*MAPT*	↓	↓	↔	↓	↑↑↑	↑↑	↓↓
*SNCA*	↑↑	↓	↔	↔	↑↑↑	↑↑	↓↓

Expression: ↑ increase; ↓ decrease; ↔ oscillation around control values; ND, no data. Genes: *MAPT,* tau protein, *LRP1*, low-density lipoprotein receptor-related protein 1, *RAGE,* receptor for advanced glycation end products, *SNCA,* α-synuclein.

In CA3, *apolipoprotein A1* (*ApoA1*) expression was decreased at 2 days, and 0.5 and 1 year, overexpressed at 7–30 days and 2 years, and close to control at 1.5 years (Table 6) (Pluta et al., 2025b; Pluta et al., 2026a). *Apolipoprotein E* (*ApoE*) overexpression was observed at 2 and 30 days, and after 1–2 years, it was decreased at 7 days and at 0.5 years it was within the control value (Table 6). *Apolipoprotein J* (*ApoJ*) was overexpressed at all times post-ischemia. In the frontal cortex, *ApoA1* expression was decreased on day 2, and at 0.5 and 1 year, increased on day 7 and at 2 years, and at the remaining times oscillated around the control level. *ApoE* expression was increased on day 2 and after 1–2 years, on day 30, it was within the control limits, and decreased in the remaining periods. *ApoJ* overexpression was on day 2 and in years 1 and 2, on day 30 and in years 1.5 it was close to control values, and in the remaining periods it was below control values (Table 6).

**TABLE 6:** Apolipoproteins A1, E and J genes expression in the CA3 area of hippocampus and frontal cortex after brain ischemia in female rats surviving from 2 days to 2 years (Pluta et al., 2025b; Pluta et al., 2026a).

Survival Genes	2 days	7 days	30 days	0.5 years	1 year	1.5 years	2 years
CA3 field							
*ApoA1*	↓	↑↑	↑	↓	↓↓	↔	↑↑
*ApoE*	↑	↓	↑	↔	↑↑↑	↑↑↑	↑
*ApoJ*	↑	↑	↑	↑	↑↑↑	↑	↑↑
Frontal cortex							
*ApoA1*	↓↓	↑	↔	↓	↓↓↓	↔	↑↑↑
*ApoE*	↑↑↑	↓	↔	↓	↑↑↑	↑↑	↑
*ApoJ*	↑↑↑	↓	↔	↓	↑↑↑	↔	↑

Expression: ↑ increase; ↓ decrease; ↔ oscillation around control values. Genes: *ApoA1,* apolipoprotein A1, *ApoE*, apolipoprotein E, *ApoJ,* apolipoprotein J.

### 4.2 Changes in Alzheimer’s disease-related mRNAs and proteins

#### 4.2.1 Amyloid precursor protein mRNAs

Following local cerebral ischemia, an increase in the mRNA of the amyloid precursor protein containing the Kunitz protease inhibitor domain was observed, whereas a decrease in the mRNA of the amyloid precursor protein 695, which lacks the Kunitz protease inhibitor domain, was revealed (Kim et al., 1998). These results indicate that focal transient cerebral ischemia affects the ratio of amyloid precursor protein containing a Kunitz protease inhibitor domain to amyloid precursor protein 695 in the cerebral cortex, and the alteration of amyloid precursor protein isoforms is likely associated with progressive neurodegeneration (Kim et al., 1998). In contrast, after irreversible focal cerebral ischemia, induction of amyloid precursor protein mRNA containing the Kunitz protease inhibitor domain was observed in the rat cerebral cortex from 1 to 21 days after injury, reaching a maximum on day 4 (Abe et al., 1991). In another study, temporal local cerebral ischemia induced amyloid precursor protein mRNA 751 and 770 within 7 days of recirculation (Koistinaho et al., 1996). Amyloid precursor protein mRNA expression was also assessed in ovariectomized female rats within 1 day after focal brain ischemia (Shi et al., 1998). One hour after a local episode of cerebral ischemia, these rats had a 68% increase in amyloid precursor protein mRNA in the penumbra. In contrast, estrogen treatment reduced the overexpression of amyloid precursor protein mRNA in this area by 26%. One day after local cerebral ischemia with ovariectomy, a 53% and 57% increase in amyloid precursor protein mRNA was found in the cerebral core and penumbra, respectively. Estrogen treatment reduced amyloid precursor protein mRNA expression in these two areas by 61% and 49%, respectively (Shi et al., 1998). These data indicate that estrogen may play a significant role in reducing amyloid precursor protein mRNA expression following focal cerebral ischemia.

In a model of regional cerebral hypoperfusion (human equivalent of silent stroke) with survival at 1, 4, and 7 days after the event, amyloid precursor protein mRNA expression was assessed (Shi et al., 2000). In these experiments, cerebral blood flow decreased by 50% on day 1 and returned to 90% on day 4. Amyloid precursor protein mRNA expression increased to 208% and 152% in the penumbra and core, respectively, 4 days after the episode and remained elevated for 7 days (Shi et al., 2000). This experiment suggests that cerebral hypoperfusion increases amyloid precursor protein mRNA expression and may contribute to amyloid formation and accumulation after silent stroke.

#### 4.2.2 Amyloid precursor protein epitopes

After cerebral ischemia with a survival of 0.5 years, staining of the brain tissue exposed the presence of N- and C-terminal of amyloid precursor protein and amyloid. Detected amyloid precursor protein fragments were noted in intracellular and extracellular spaces (Pluta et al., 1994a; Hall et al., 1995; Tomimoto et al., 1995; Ishimaru et al., 1996a; Yokota et al., 1996; Pluta, 1997; Pluta et al., 1997a; Pluta et al., 1998; Lin et al., 1999; Pluta, 2000a; Pluta, 2000b; Lin et al., 2001; Sinigaglia-Coimbra et al., 2002; Fujioka et al., 2003; Pluta et al., 2009; Jabłoński et al., 2011; Pluta and Jabłoński, 2012; Pluta et al., 2012a). The presence of stained fragments of amyloid precursor protein was found in neuronal and neuroglial cells (Banati et al., 1995; Palacios et al., 1995; Pluta et al., 1997a; Pluta, 2000a; Pluta, 2000b; Nihashi et al., 2001; Badan et al., 2003; Badan et al., 2004; Pluta et al., 2009). However, when survival was 0.5–1 year, staining of brain tissue was observed only for C-terminal of amyloid precursor protein and amyloid (Pluta et al., 1998; Pluta, 2000a; Pluta et al., 2009; Jabłoński et al., 2011). The unusual buildup of amyloid in the cytoplasm of reactive astrocytes suggests their involvement in the complex repair of brain tissue post-ischemia, contributing at the same time to their death (Pluta et al., 1994a; Pluta, 2000a; Pluta, 2000b; Wyss-Coray et al., 2003; Takuma et al., 2004). Furthermore, it has been suggested that astrocytes with high accumulation of various parts of the amyloid precursor protein may be involved in the development of the glial scar (Pluta, 2000a; Pluta, 2000b; Nihashi et al., 2001; Badan et al., 2003; Badan et al., 2004).

In the subcortical and periventricular white matter after cerebral ischemia, staining for all fragments of amyloid precursor protein was observed (Pluta et al., 2006; Pluta, 2007; Pluta et al., 2008; Pluta et al., 2009). It was found that the more severe the white matter damage post-ischemia, the more extensive the staining for all amyloid precursor protein fragments in the extracellular space (Yam et al., 1997). It is believed that this type of changes is responsible for the appearance of leukoaraiosis after an episode of brain ischemia (Pluta et al., 2008). The extracellular deposits of all amyloid precursor protein fragments ranged from very small dots, through diffuse amyloid plaques, to dense deposits resembling senile amyloid plaques (Pluta et al., 1994a; Pluta et al., 1998; Pluta, 2000a; Pluta, 2000b; Pluta et al., 2000; Pluta, 2002a; Pluta, 2002b; Pluta, 2003; Pluta et al., 2003; Pluta, 2005; van Groen et al., 2005; Pluta, 2007; Pluta et al., 2009; Pluta et al., 2010). Multifocal diffuse post-ischemic amyloid plaques predominated in the cerebral cortex, hippocampus, entorhinal cortex, corpus callosum, and around the lateral ventricles. Dense deposits resembling senile amyloid plaques were found in the thalamus (van Groen et al., 2005).

The presence of all amyloid precursor protein fragments, including amyloid in neurons as well as in neuroglial cells, suggests an important role of amyloid precursor protein epitopes in the progression of neurodegeneration post-ischemia (Pluta et al., 1994a; Yokota et al., 1996; Pluta, 2002a; Pluta, 2002b; Badan et al., 2003; Badan et al., 2004). Furthermore, amyloid accumulation in synapses may cause their destruction and this may trigger retrograde neuronal death after ischemia (Oster-Granite et al., 1996). Thus, the process of accumulation of amyloid precursor protein epitopes after ischemia may be responsible for secondary neurodegenerative phenomena that may worsen the prognosis after ischemia through continued neuronal death (Pluta et al., 1997a; Pluta et al., 1998; Pluta et al., 2009; Jabłoński et al., 2011; Kiryk et al., 2011; Pluta et al., 2011; Pluta and Jabłoński, 2012; Pluta et al., 2012a; Pluta et al., 2012b). Amyloid is believed to result from damage and death of neurons due to cerebral ischemia (Ishimaru et al., 1996a). Due to its neurotoxic properties, amyloid, following ischemia of neuronal and neuroglial cells, triggers additional intracellular mechanisms leading to their damage and/or death (Giulian et al., 1995; Cotter et al., 1999; Pluta et al., 2012a).

#### 4.2.3 Amyloid precursor protein processing secretases mRNA

Cleavage of amyloid precursor protein by α-secretase does not lead to the amyloidogenic pathway. As a result of cerebral ischemia in animals, a decrease in the mRNA expression of α-secretase and degrading enzymes such as neprilysin and endothelin-converting enzyme was observed in the hippocampus, cerebral cortex and striatum (Nalivaeva et al., 2004; Yan et al., 2007). Furthermore, it was revealed that hypoxic preconditioning triggered the restoration of α-secretase, neprilysin, and endothelin-converting enzyme mRNA expression (Nalivaeva et al., 2004).

In the amyloidogenic pathway, amyloid precursor protein is metabolized by β- and γ-secretases, leading to the formation of the neurotoxic β-amyloid peptide (Pluta et al., 2013a; Pluta et al., 2013b). β-Secretase mRNA expression was elevated 1 day after injury and persisted for up to 3 days in the hippocampus and cerebral cortex compared to control animals (Blasko et al., 2004). Above changes in β-secretase mRNA occurred in neuronal and neuroglial cells. Changes in β-secretase mRNA expression in the hippocampus and cerebral cortex were accompanied by an increase in protein levels. β-Secretase activity, measured by the generation of amyloid, a cleavage product of the amyloid precursor protein, was elevated for 2 days and returned to baseline levels 7 days after injury (Blasko et al., 2004).

β-Secretase and presenilin 1 proteins have been shown to co-accumulate in swollen axons within 6 months after ischemia (Chen et al., 2004). In another study, cerebral ischemia in rats resulted in decreased β-secretase protein levels 1 month after ischemia (Chuang et al., 2008). In contrast, reversible focal cerebral ischemia in female rats resulted in a 30% increase in β-secretase activity and a 67% increase in its protein level in the neocortex compared to the contralateral cortex (Wen et al., 2004a). In the case of middle cerebral artery occlusion in rats, increased levels of β-secretase mRNA and protein were noted in the striatum (Xiong et al., 2008). Moreover, after reversible brain ischemia, overexpression of β-secretase, cathepsin B and glutaminyl cyclase mRNA was noted in brain parenchyma (Ye et al., 2009). This indicates that all three mRNAs associated with amyloid production may be involved in the acute and chronic metabolism of amyloid precursor proteins after reversible ischemic brain injury and will be helpful in understanding the processes underlying the association of brain ischemia with the development of Alzheimer’s disease (Ye et al., 2009).

mRNA of presenilin’s 1 and 2, which, when induced by transient cerebral ischemia, participate in the generation of amyloid via the γ-secretase complex (Tanimukai et al., 1998; Pennypacker et al., 1999). Within 1–3 days of reperfusion, overexpression of presenilin 1 mRNA was revealed in CA3 and the dentate gyrus neurons, areas relatively resistant to ischemia (Tanimukai et al., 1998). Another study also showed an increase in presenilin 1 and 2 mRNA expression in the cerebral cortex, hippocampus, and striatum on days 4 and 8 after local cerebral ischemia (Pennypacker et al., 1999). The maximum expression of presenilin’s mRNA was found in the cerebral cortex, with a 7–10-fold increase. It should be noted that overexpression was observed mainly on the contralateral side to focal cerebral ischemia. This phenomenon can be explained by the loss of presenilin mRNA-expressing cells on the ipsilateral side.

#### 4.2.4 Presenilins

Staining for presenilin’s was more intense in neuroglial cells than in neurons and was clearly visible in the trace of pyramidal neurons in the CA1 area of the hippocampus after cerebral ischemia (Pluta, 2001). Presenilin 1 has been shown to increase the sensitivity of neuronal cells to ischemic episodes by increasing intracellular calcium concentration (Mattson et al., 2000; Pluta et al., 2009). Furthermore, studies have shown that presenilin 1 and increased intracellular calcium levels control glutamate uptake by neurons (Yang et al., 2004; Pluta et al., 2009). Together, these results indicate that presenilin, in combination with intracellular calcium, may control glutamate neurotoxicity in the post-ischemic brain.

#### 4.2.5 Apolipoproteins mRNA

Seven days after ischemia, apolipoprotein E mRNA overexpression (3-fold increase) was found in the hippocampus of gerbils, which is likely associated with post-ischemic amyloidogenesis and neuronal death (Ali et al., 1996). However, after transient focal brain ischemia in rats, apolipoprotein E mRNA expression was noted in the penumbra, reaching a maximum on days 7 and 21 after injury (Kamada et al., 2003). Whereas in the ischemic core, the maximum increase in apolipoprotein E mRNA expression was detected 7 days after ischemia (Kamada et al., 2003). After persistent local brain ischemia, overexpression of apolipoprotein J mRNA was observed in the penumbra, with the peak expression occurring 3 days later (Van Beek et al., 2000). It has been suggested that overexpression of apolipoprotein J mRNA after brain ischemia may trigger neuroinflammation, which is an important component in secondary brain damage (Van Beek et al., 2000).

#### 4.2.6 Apolipoproteins

After ischemic brain injury, a time-dependent accumulation of apolipoproteins A1, E, and J was observed in pyramidal neurons of the CA1 and CA2 regions of the hippocampus (Kida et al., 1995). The presence of these apolipoproteins has been demonstrated in neurons condemned to death by apoptosis (Kida et al., 1995). The presence of apolipoproteins A1, E, and J is thought to be a result of delayed neuronal death rather than being related to the events that cause it (Walton et al., 1996).

At different survival times post-ischemia, strong staining of apolipoproteins A1, E and J was found in the intra- and extracellular spaces (Hall et al., 1995; Kida et al., 1995; Ishimaru et al., 1996b; Pluta, 2000a; Kamada et al., 2003). Intracellular staining was found in both partially and completely damaged neuronal cells due to brain ischemia (Kida et al., 1995; Pluta, 2000a). It should be noted that staining of apolipoproteins A1, E, and J in neuroglial cells was less frequent and less intense after ischemia (Kamada et al., 2003). Clearly demarcated, scattered extracellular apolipoprotein deposits were also revealed. Intense staining was also found in acellular, irregular, spider-shaped necrotic foci after cerebral ischemia, mainly in the hippocampus (Kida et al., 1995; Ishimaru et al., 1996a). It should be noted that apolipoproteins A1, E and J also accumulated at sites of amyloid deposition (Kida et al., 1995). Apolipoprotein E is believed to influence the transformation of soluble amyloid into oligomeric and fibrillar forms. Apolipoprotein J, on the other hand, supports the transport of amyloid peptides across the ischemic blood-brain barrier. Apolipoprotein E is believed to enhance the apoptotic effects of amyloid in neurons (Ji et al., 2002).

In summary, apolipoproteins A1, E, and J influence neurotoxicity, structure, and amyloid accumulation after ischemia. Apolipoproteins E and J are also thought to be involved in the production of amyloid prior to its deposition. The above information indicates that apolipoproteins A1, E and J play a key role in the control of amyloid metabolism in the extracellular space, regardless of its production after ischemia. These data indicate that the accumulation of apolipoproteins A1, E, and J after ischemia may be a secondary consequence of injury and may impair the healing of ischemic neurons and the brain as a whole.

### 4.3 Other proteins

#### 4.3.1 Tau protein

Following cerebral ischemia, strong staining of tau protein was discovered in neurons and neuroglial cells in hippocampus and cortex (Dewar et al., 1993; Dewar et al., 1994; Geddes et al., 1994; Dewar and Dawson, 1995; Irving et al., 1997; Sinigaglia-Coimbra et al., 2002; Uchihara et al., 2004; Majd et al., 2016a; Majd et al., 2016b; Majd et al., 2016c; Fujii et al., 2017). Another study showed that tau protein can inhibit the transport of amyloid precursor protein from the neuronal body to the axons and dendrites, resulting in the accumulation of amyloid precursor protein in the neuronal body (Stamer et al., 2002). Moreover, available studies have revealed that after ischemia, hyperphosphorylated tau protein is observed in neurons of cortex, which accompanies their apoptosis (Wen et al., 2004b; Wen et al., 2004c; Wen et al., 2007; Majd et al., 2016a; Majd et al., 2016b; Majd et al., 2016c; Fujii et al., 2017). The above observations indicate that apoptosis of neuronal cells after cerebral ischemia is directly related to hyperphosphorylation of tau protein. Furthermore, cerebral ischemia has been shown to lead to the accumulation of hyperphosphorylated tau protein in the form of paired helical filaments, which are the main component of neurofibrillary tangles in Alzheimer’s disease (Khan et al., 2018). It has also been presented that transient focal cerebral ischemia was associated with the formation of neurofibrillary tangle-like (Wen et al., 2004b; Wen et al., 2004c; Wen et al., 2007).

#### 4.3.2 α-Synuclein

Experimental cerebral ischemia has been shown to cause the accumulation of α-synuclein in the presynaptic part of hippocampal synapses (Ishimaru et al., 1998; Kitamura et al., 2001). Moreover, the presence of α-synuclein has been demonstrated in neuroglial cells in the degenerated hippocampus after ischemia (Ishimaru et al., 1998). At 7 days post-ischemia, strong staining for α-synuclein was also observed perivascularly in the CA1 area of the hippocampus (Kitamura et al., 2001). α-Synuclein, which influences synaptic function, may additionally cause retrograde neuronal death after ischemia (Goedert, 2001), which consequently leads to the development of cognitive impairments (Hashimoto and Masliah, 1999).

### 4.4 Neurodegenerative changes

#### 4.4.1 Neuropathophysiology

After ischemia, massive release of glutamate into the extracellular space and intracellular calcium overload were observed (Pluta et al., 1988; Łazarewicz et al., 1989; Salinska et al., 1989; Łazarewicz et al., 1990; Pluta et al., 1990; Pluta et al., 1991b; Salińska et al., 1991; Łazarewicz et al., 1993). Intracellularly, phospholipases, endonucleases, nitric oxide synthase, and proteases are activated by calcium, and the end result of this process is damage to membranes, the nucleus, and cytoplasmic organelles, leading to neuronal necrotic death (Pluta et al., 2022). Necrosis develops as a result of loss of energy and osmotic homeostasis, affecting the vast majority of neuronal cells in brain parenchyma (Dong et al., 1997; Rathmell and Thompson, 1999; Pluta et al., 2022). The death of neurons by apoptosis is determined by the duration of cerebral ischemia (Nitatori et al., 1995). Two main mechanisms have been described to trigger apoptosis in ischemic neurons: receptor-mediated apoptosis and mitochondrial apoptosis (Fujimura et al., 1998; Sugawara et al., 1999). After cerebral ischemia, caspase 3 plays a key role in apoptotic neuronal death (Sugawara et al., 2004; Ułamek-Kozioł et al., 2016; Pluta et al., 2017; Ułamek-Kozioł et al., 2017; Ułamek-Kozioł et al., 2019; Pluta et al., 2024; Pluta et al., 2025a). It should also be noted that autophagy and mitophagy are related to apoptosis (Rosenbaum et al., 2000; Ułamek-Kozioł et al., 2016; Pluta et al., 2017; Ułamek-Kozioł et al., 2017; Ułamek-Kozioł et al., 2019; Pluta et al., 2024; Pluta et al., 2025a). In addition, another neuronal death pathway, called necroptosis, has been revealed, which also occurs after brain damage caused by ischemia (Unal-Cevik et al., 2004; Degterev et al., 2005). Another process of neuronal death is autophagy-programmed death which plays an important role in post-ischemic cerebral pathology (Tsujimoto and Shimizu, 2005; Adhami et al., 2007; Ułamek-Kozioł et al., 2013; Ułamek-Kozioł et al., 2016; Pluta et al., 2017; Ułamek-Kozioł et al., 2017; Wang et al., 2018; Ułamek-Kozioł et al., 2019; Pluta et al., 2024; Pluta et al., 2025a; Pluta and Ułamek-Kozioł, 2026). In addition to acute or chronic neuronal death, a decrease in acetylcholine levels has been observed in the brain following ischemia (Yuan et al., 2020; Li B. et al., 2023).

#### 4.4.2 Neuropathology

Damage resulting from focal and global cerebral ischemia mainly affects the hippocampus (Kirino, 1982; Pulsinelli et al., 1982; Smith et al., 1984; Pluta et al., 1991a; Pluta, 2000a; Pluta, 2002a; Pluta, 2002b; Pluta et al., 2009), which is responsible for memory and learning, similarly to Alzheimer’s disease. In the hippocampus, selective death of pyramidal neurons occurs 2–7 days after ischemia and is called delayed neuronal death (Kirino, 1982; Pulsinelli et al., 1982; Pluta, 2000a; Pluta, 2002a; Pluta, 2002b; Pluta et al., 2009). Changes in the striatum are observed mainly in the dorsolateral region and concern medium-sized neurons, while in the brain cortex changes occur in layers 3, 5 and 6 (Pulsinelli et al., 1982; Pluta, 2000a; Pluta, 2002a; Pluta, 2002b). Furthermore, prolonged survival after brain ischemia, lasting up to 2 years, has been shown to result in changes in neuronal cells in hippocampal regions that are not selectively sensitive to ischemia (Pluta et al., 2009). In the CA2, CA3 and CA4 sectors of the hippocampus, alterations characteristic of early (acute) changes after ischemia were observed (Pluta et al., 2009). Chronic neuronal damage, predominant in the early phase after ischemia, was also observed in the brain after long-term survival of animals (Pluta et al., 2009).

#### 4.4.3 Blood-brain barrier

An ischemic episode causes a chronic increase in the permeability of the blood-brain barrier to cellular and non-cellular blood components (Mossakowski et al., 1993; Mossakowski et al., 1994; Pluta et al., 1994c; Wisniewski et al., 1995; Shinnou et al., 1998; Ueno et al., 2002; Pluta, 2003; Pluta et al., 2023b). In the case of post-ischemic blood-brain barrier damage, two unusual and characteristic features should be noted. One is important due to the chronic effects of neurotoxic amyloid extravasation, contributing to irreversible neurodegeneration, and the other concerns the leakage of blood cellular elements, e.g., platelets, which causes acute, massive, and mechanical destruction of brain tissue (Hallenbeck et al., 1986; Pluta et al., 1994b; Pluta et al., 1996; Pluta et al., 1997b; Pluta, 2005; Jabłoński et al., 2011; Pluta et al., 2021a; Pluta et al., 2021b; Pluta et al., 2023b). The ability of amyloid to cross a damaged blood-brain barrier may lead to local neurotoxic effects on certain neuronal populations, which may increase amyloid production and accumulation in brain tissue (Pluta et al., 2003; Pluta et al., 2006; Pluta et al., 2023b).

#### 4.4.4 Cerebral amyloid angiopathy

Blood-brain barrier permeability following ischemia may initiate a continuous process of circulating amyloid accumulation in the wall of cerebral blood vessels (Pluta et al., 1996; Pluta et al., 1997b; Pluta, 2003; Pluta et al., 2003; Pluta et al., 2023b), a phenomenon called cerebral amyloid angiopathy (Pluta et al., 2021a; Pluta et al., 2021b; Rost et al., 2022; Pluta, 2025b). Collagen accumulation and basement membrane thickening following brain ischemia contribute to amyloid deposition in the blood vessel wall (Pluta et al., 1994c; Pluta et al., 2021a; Pluta et al., 2021b). Furthermore, the initial accumulation of amyloid in the vascular wall post-ischemia may further cause degeneration of endothelial cells and pericytes, which negatively affects the activity of the blood-brain barrier. In turn, blood-brain barrier dysfunction leads to the incorporation of circulating amyloid from the blood and interstitial fluid, which causes further amyloid accumulation and, consequently, the development of cerebral amyloid angiopathy and ultimately irreversible vascular degeneration. Under such conditions, amyloid from blood and interstitial fluid can also interact with the inner and outer parts of the capillary wall (Pluta et al., 2023b). Additionally, microbleeds in brain parenchyma have been described in animals’ post-ischemia (Hossmann et al., 1980; Pluta, 1985; Chen and Ye, 2022). These small vascular hemorrhages then recruit and activate platelets near the rupture. When activated, platelets release biologically active molecules at the site of blood vessel damage that affect the functioning of the vessel wall (Pluta et al., 1994b; Stokes and Granger, 2012; Kniewallner et al., 2015). It is important to note that platelets contain very large amounts of the amyloid precursor protein, which generates amyloid, as well as amyloid itself. The smaller, 40-amino acid form of amyloid predominates in platelets (Kniewallner et al., 2015). It has been suggested that this peptide, as in the case of cerebral amyloid angiopathy in Alzheimer’s disease, may accumulate and contribute to embolism at the site of injury (Pluta et al., 1994b). It is currently believed that these accumulations of blood-derived amyloid and platelets in the damaged vascular wall in the post-ischemic period may be a factor in the development of cerebral amyloid angiopathy (Pluta et al., 1994b; Rost et al., 2022; Pluta et al., 2023b; Pluta, 2025b). This phenomenon presumably operates as a vicious circle after ischemic injury.

#### 4.4.5 Neuroinflammation

Strong neuroglial cells response was observed in areas affected by ischemic neuronal cell changes (Petito et al., 1990; Schmidt-Kastner et al., 1990; Gehrmann et al., 1992; Morioka et al., 1992; Orzyłowska et al., 1999; Pluta, 2000a; Pluta, 2002a; Pluta, 2002b; Sekeljic et al., 2012; Radenovic et al., 2020). Intensive cytokines, e.g., interleukin-1β staining was demonstrated in astrocytes in acute phase post-ischemia in the CA1 region of the hippocampus (Orzyłowska et al., 1999; Touzani et al., 2002; Pluta, 2025b; Pluta, 2025c). Interleukin-1β has been shown to play a key role in neuronal damage and the development of edema following cerebral ischemia (Yamasaki et al., 1995). In cerebral ischemia and Alzheimer’s disease, interleukin-1 stimulates neurons to amyloidogenic metabolism of amyloid precursor protein and this triggers the release of neuroinflammatory mediators and a vicious cycle (Pluta, 2025b; Pluta, 2025c).

Immunohistochemical studies performed 1–2 years after brain ischemia using the microglia marker Iba1 and the astrocyte marker GFAP revealed a cellular inflammatory response (Sekeljic et al., 2012; Radenovic et al., 2020; Pluta, 2025b; Pluta, 2025c). The study presented significant astrocyte activation in the following brain regions: CA1 and CA3 of the hippocampus and dentate gyrus, motor and sensorimotor cortex, striatum, and thalamus, whereas microglial activation was found only in CA1, CA3, and the motor cortex (Sekeljic et al., 2012; Radenovic et al., 2020; Pluta, 2025b; Pluta, 2025c). In particularly sensitive brain regions, microglia and astrocytes simultaneously showed significant activation, whereas in resistant brain regions only astrocytes were activated. This revealed less intense neuroinflammation in ischemia-resistant brain regions. These neuroinflammatory processes were supported by microglial and astrocyte activity up to 2 years after brain ischemia (Sekeljic et al., 2012; Radenovic et al., 2020; Pluta, 2025b; Pluta, 2025c). The study demonstrated a chronic effect of brain ischemia on the neuroinflammatory response over the 2-year period. The research results indicate that microglia and astrocytes are not only witnesses but also active and important participants in post-ischemic brain neurodegeneration. Studies have revealed a significant role for neuroinflammation in whole-brain neurodegeneration following ischemia. Chronic accumulation of neuroinflammatory factors in the brain due to ischemia may activate a self-sustaining cycle that transforms ischemic pathology into the neurodegeneration characteristic of Alzheimer’s disease. This role is complex and requires further investigation. Evidence has shown that the effect of cerebral ischemia on microglia and astrocyte activity varies significantly across brain structures. This partially explains why the severity of neurodegenerative changes in the post-ischemic brain varies significantly across regions and does not develop simultaneously (Pluta, 2024).

#### 4.4.6 Synaptic alterations

In the rat hippocampus after cerebral ischemia, a decrease in the levels of both postsynaptic density protein 95 and synaptophysin was observed (Wang et al., 2010; Zhao et al., 2014). Moreover, changes in the synaptic ultrastructure were observed in the CA1 region of the hippocampus (Neumann et al., 2013). Other studies indicate that cerebral ischemia stimulates synaptic autophagy, which is presumably associated with neuronal death in the CA1 area of the hippocampus post-ischemia (Ruan et al., 2012; Ułamek-Kozioł et al., 2013; Ułamek-Kozioł et al., 2017). Persistent and isolated synaptic dysfunction resulting from experimental transient cerebral ischemia has been demonstrated (Hofmeijer and van Putten, 2012; Neumann et al., 2013). Additionally, after cerebral ischemia, a reduction in excitatory synaptic transmission in the CA1 subfield of the hippocampus was observed (Pluta et al., 1988; Łazarewicz et al., 1989; Salinska et al., 1989; Łazarewicz et al., 1990; Pluta et al., 1990; Pluta et al., 1991b; Salińska et al., 1991; Łazarewicz et al., 1993). The ischemia-induced increase in intracellular calcium concentration enhances calpain function in neurons, and calpain target proteins are present in glutamatergic and GABAergic synapses. In the case of brain damage caused by ischemia, calpain cleaves pre- and postsynaptic proteins, which contributes to the death of neurons in post-ischemic brain parenchyma (Curcio et al., 2016).

#### 4.4.7 White matter lesion

Following an episode of cerebral ischemia, changes in the white matter and activation of neuroglial cells have been observed (Pluta, 2000a; Pluta, 2002a; Pluta, 2002b; Fernando et al., 2006; Pluta et al., 2006; Pluta et al., 2008; Pluta et al., 2009; Scherr et al., 2012; Sekeljic et al., 2012; Thiebaut de Schotten et al., 2014; Zamboni et al., 2017; Radenovic et al., 2020). Cerebral ischemia in rats causes more severe white matter lesion in the corpus callosum and subcortical white matter (Wakita et al., 1994; Pluta et al., 2006; Pluta et al., 2008; Pluta et al., 2009). These observations are consistent with increased activation of neuroglial cells in the corpus callosum post-ischemia (Yoshizaki et al., 2008). Cerebral ischemia causes, among other things, increased permeability of the blood-brain barrier in the white matter, which causes the passage of inflammatory cells and β-amyloid peptide from the blood to the white matter, intensifying its damage (Pluta et al., 1996; Pluta et al., 1997b; Pluta et al., 1999; Pluta et al., 2000; Anfuso et al., 2004; Lee et al., 2005; Zetterberg et al., 2011).

#### 4.4.8 Generalized brain atrophy

Evidence suggest that transient ischemic brain injury causes widespread neuronal loss in structures that are selectively sensitive and insensitive to ischemia (Pluta, 2000a; Pluta et al., 2009). Changes following cerebral ischemia progress gradually and are closely related to the time of observation (Pluta et al., 2009). These processes are characteristic not only for early changes after cerebral ischemia, but also for changes in the late stages after ischemia (Pluta et al., 2009). Several years after ischemia, neuropathological processes lead to generalized brain atrophy (Hossmann et al., 1987; Pluta, 2000a; Pluta et al., 2009; Jabłoński et al., 2011). Gross examination of the brain performed up to 2 years post-ischemia revealed hydrocephalus, characteristic of atrophy (Hossmann et al., 1987; Pluta, 2000a; Pluta et al., 2009; Jabłoński et al., 2011). There was also an increase in the subarachnoid space around the cerebral hemispheres (Pluta, 2000a). In addition, atrophy of the hippocampus and striatum was observed (Pluta, 2000a; Pluta et al., 2009; Jabłoński et al., 2011). The cerebral cortex post-ischemia was narrow, indicating artificially increased neuronal density (Pluta, 2000a; Pluta et al., 2009). An additional element intensifying the atrophy of the brain parenchyma were diffuse changes in the white matter, taking the form of cavitations and rarefaction, indicating advanced spongiosis (Pluta, 2000a; Pluta et al., 2009). This phenomenon can be explained by a significant loss of neurons and a simultaneous increase in the permeability of the blood-brain barrier, occurring in both the early and late stages after cerebral ischemia (Pluta et al., 1994c; Pluta, 2003; Pluta, 2005; Pluta et al., 2010).

### 4.5 Dementia

Neuronal damage and/or loss due to ischemia and recirculation also resulted in persistent behavioral changes (Block, 1999; de la Tremblaye and Plamondon, 2011; Kiryk et al., 2011; Li et al., 2011; Pluta et al., 2011; Cohan et al., 2015; Liu et al., 2026). Ischemic-recirculation injury does not cause long-term neurological deficits in animals (Block, 1999). During the post-ischemic recirculation period, spontaneous recovery of sensorimotor activity was observed (Kiryk et al., 2011). Following brain lesions caused by ischemia and reperfusion, animals have been observed to exhibit excessive locomotor activity (Kuroiwa et al., 1991; Karasawa et al., 1994), the same type as in patients with Alzheimer’s disease. The hyperactivity was caused by the death of pyramidal neurons in the hippocampus (Kuroiwa et al., 1991). Longer ischemic time and consequently longer duration of locomotor hyperactivity correlated directly with increased number of hippocampal neurons lost and neuroinflammation (Block, 1999; Langdon et al., 2008; Pluta et al., 2010; Sekeljic et al., 2012; Radenovic et al., 2020; Pluta, 2025b; Pluta, 2025c). After brain damage due to ischemia, habit disorders were observed, manifesting themselves by prolonged examination time (Mileson and Schwartz, 1991; Colbourne and Corbett, 1995). In addition, cerebral ischemia causes a deficit in reference and working memory (Davis et al., 1986; Kiyota et al., 1991; Kiryk et al., 2011). Brain ischemia-reperfusion injury in experimental animals slowly leads to spatial memory deficits during the recirculation period (Block and Schwarz, 1998; Karhunen et al., 2003; Kiryk et al., 2011). Cognitive deficits progressed with increasing recirculation time (Roof et al., 2001; Karhunen et al., 2003; Kiryk et al., 2011). Furthermore, repeated transient ischemic brain injuries in animals have shown persistent locomotor hyperactivity, durable cognitive deficits, and reduced anxiety levels (Ishibashi et al., 2006). The above-mentioned behavioral abnormalities were associated with massive brain atrophy (Hossmann et al., 1987; Pluta, 2000a; Pluta, 2002a; Pluta et al., 2009; Jabłoński et al., 2011; Pluta et al., 2012a; Pluta et al., 2012b), neuronal cells death in the CA1 area of hippocampus, caudate nucleus, brain cortex (Ishibashi et al., 2006; Pluta et al., 2009; Pluta et al., 2010; Pluta et al., 2012b), amygdala and perirhinal cortex (de la Tremblaye and Plamondon, 2011). Alertness and sensory-motor skills deteriorate within 1–2 days and are reversible, whereas learning and memory deficits progress irreversibly slowly and persist forever (Langdon et al., 2008; Kiryk et al., 2011; Liu et al., 2026).

## 5 Hypothetical ischemic pattern of Alzheimer’s disease development

Alzheimer’s disease is characterized by progressive, massive neuronal loss, blood-brain barrier changes, neuroinflammation, deposition of extracellular amyloid plaques, intracellular neurofibrillary tangles, and cerebral amyloid angiopathy. In the brains of Alzheimer’s disease patients, there is a direct link between widespread amyloid deposition, the accumulation of neurofibrillary tangles, and structures that are irreversibly damaged (Fonte et al., 2001; Klunk et al., 2004). On the other hand, the amount of amyloid in the brain does not directly correlate with the duration of Alzheimer’s disease (Hyman et al., 1993). Although it is well known that the degree of neuronal loss correlates positively with the severity and intensification of dementia, the mechanism leading to their death remains unclear (Gomez-Isla et al., 1997). The notion that amyloid accumulation contributes partially or completely, if at all, to the development of massive neuronal death in Alzheimer’s disease remains controversial in the international literature (Kienlen-Campard et al., 2002; Selkoe, 2002; Meyer-Luehmann et al., 2003; Szabo, 2025). Other controversial observations suggest that neuronal death coincides with the formation of neurofibrillary tangles, which are composed of hyperphosphorylated tau protein (Andorfer et al., 2005). In contrast, a study in a transgenic model of Alzheimer’s disease showed that the process of neuronal death does not correlate with the tau protein modifications within the individual neuron that is destined to die, suggesting that neuronal death may occur independently of the development of neurofibrillary tangles (Andorfer et al., 2005). Moreover, there is data supporting the above observations that some neurons in Alzheimer’s disease may die without forming neurofibrillary tangles (Gomez-Isla et al., 1997). It can therefore be concluded that there is no relationship between neurofibrillary tangles, amyloid plaques, neuronal death and Alzheimer’s disease dementia, and that neurofibrillary tangles and amyloid plaques may occur as independent phenomena and be the result of degenerative processes, not their cause (Armstrong, 2006). On the other hand, various studies have shown that up to 10% of patients with advanced Alzheimer’s disease do not have amyloid plaques (Jellinger and Attems, 2007; Nelson et al., 2009; Jicha et al., 2012; Andrade-Moraes et al., 2013; Kovacs et al., 2013; Crary et al., 2014). These observations provide additional evidence that neuronal death may not be a direct and/or primary consequence of amyloid plaques and neurofibrillary tangles formation, but rather may be a consequence of other global neuropathology, e.g., cerebral ischemia. Another important element of neuropathology in Alzheimer’s disease is the deposition of amyloid in the walls of small blood vessels in the brain. The accumulation of amyloid in the walls of small cerebral vessels causes pathological alterations in the neurovascular network and/or results in dysfunction of the blood-brain barrier and local no-reflow phenomenon. The insufficiency of barrier causes serum amyloid to leak into the surrounding brain parenchyma (Pluta et al., 1996; Pluta et al., 1997b; Pluta et al., 1999; Pluta et al., 2000; Lee et al., 2005; Zetterberg et al., 2011). Some evidence indicates that about 80% of the amyloid plaques in the transgenic model of Alzheimer’s disease (Dickstein et al., 2006) and about 90% of human amyloid plaques are in close contact with blood-brain barrier vessels (Kawai et al., 1990).

Study of different types of amyloid plaques using serial brain sections from Alzheimer’s disease patients using light and electron microscopy revealed an association between amyloid plaques and microvessels. The cores of amyloid plaques form tight contact with microvessels, and spreading amyloid is visible in the surrounding brain parenchyma. The presence of immunoglobulins and complement factor was detected in the molecular composition of the core of amyloid plaques (Armstrong, 2006). Confocal laser scanning and scanning electron microscopy have shown a close relationship between amyloid buildup and the neurovascular system, particularly with β-amyloid peptide 1–40. Moreover, a study conducted using confocal laser scanning microscopy showed that deposits of β-amyloid 1–40 peptide are deposited not only in the walls of cerebral vessels but also around them (Miyakawa and Kuramoto, 1989; Miyakawa et al., 2000). These observations seem to indicate that blood-brain barrier dysfunction may exacerbate the abnormal transport of β-amyloid peptide 1–40 from the blood into the brain parenchyma in people with Alzheimer’s disease (Pluta et al., 1996; Miyakawa, 2002). In contrast, in transgenic mice with increased blood levels of β-amyloid peptide, no β-amyloid peptide deposits were detected in the brain (Fukuchi et al., 1996).

The evidence of no difference in the levels of β-amyloid peptides 1–40 and 1–42 in the blood of patients with sporadic Alzheimer’s disease (Seubert et al., 1992) and the finding of deposits of these peptides in brain parenchyma support the hypothesis that the blood-brain barrier must be damaged resulting in increased transport of amyloid from the blood into the brain tissue in these patients (Miyakawa et al., 2000). The observed amyloid-induced degeneration of capillaries and arteries indicates that the core of amyloid plaques may consist of materials derived from the circulatory system. Therefore, the passage and accumulation of amyloid from the blood into the surrounding brain parenchyma and its deposition in the walls of blood vessels indicate pathological changes in the blood-brain barrier (Pluta et al., 1996). Cerebral ischemia, which is associated with the development of Alzheimer’s disease, is known to affect the integrity of the blood-brain barrier, which may lead to increased translocation of amyloid from the blood into the surrounding brain tissue (Pluta et al., 1994a; Pluta et al., 1996; Pluta et al., 1997b; Pluta et al., 1999; Pluta et al., 2000). For this reason, a priority should be to investigate the role of ischemic factor in the development of sporadic Alzheimer’s disease. Furthermore, controversy persists as to whether post-ischemic dementia is a distinct disease entity, from Alzheimer’s disease dementia, or simply two extreme descriptions of the same clinical condition. A significant and growing body of evidence indicates the presence of ischemic processes in Alzheimer’s disease (Pluta et al., 1994b; Pluta et al., 1996; Kalaria, 2000; Pluta et al., 2009; Pluta et al., 2013a; Pluta et al., 2013b; Kocki et al., 2015; Pluta et al., 2016a; Pluta et al., 2016b; Ułamek-Kozioł et al., 2016; Salminen et al., 2017; Ułamek-Kozioł et al., 2017; Pluta et al., 2018b; Ułamek-Kozioł et al., 2019; Lecordier et al., 2022; Zhang et al., 2024; Pluta, 2025a; Liu et al., 2026; Pluta and Ułamek-Kozioł, 2026).

Cerebral ischemia has also been found to be a factor lowering the death threshold of neuronal cells (Koistinaho et al., 2002). Some studies in a transgenic animal model of Alzheimer’s disease suggest that hippocampal neuronal death, a common hallmark of Alzheimer’s disease, is not amyloid-dependent (Games et al., 1995; Schmitz et al., 2004; Armstrong, 2006). Other studies have shown that amyloid is formed in response to ischemic neuronal damage as a result of amyloidogenic metabolism of amyloid precursor protein stimulated by ischemia (Ishimaru et al., 1996a; Kocki et al., 2015; Pluta et al., 2016a; Pluta et al., 2016b). In cases of ischemic brain damage in humans, the presence of amyloid has been observed in neuronal bodies and in degenerated neurites, similar to that seen in Alzheimer’s disease (Jendroska et al., 1995; Wisniewski and Maslinska, 1996; Jendroska et al., 1997; Qi et al., 2007; Maślińska et al., 2011). Tau protein modifications and neurofibrillary tangles may also be part of the neuronal response to cerebral ischemia (Kato et al., 1988; Sinigaglia-Coimbra et al., 2002; Wen et al., 2004a; Wen et al., 2004b; Wen et al., 2007; Majd et al., 2016a; Majd et al., 2016b; Majd et al., 2016c; Bi et al., 2017; Fujii et al., 2017; Tuo et al., 2017; Basurto-Islas et al., 2018; Khan et al., 2018; Pluta et al., 2018a; Pluta et al., 2018b; Hatsuta et al., 2019). Experimental brain ischemia-reperfusion injury also causes overexpression of the amyloid precursor protein gene, e.g., in the hippocampus and cerebral cortex, suggesting that amyloid precursor protein gene induction may be a characteristic response to loss of neuronal activity (Kocki et al., 2015; Pluta et al., 2016a). In support of this conclusion, staining for different amyloid precursor protein epitopes was observed in ischemic neuronal bodies and dystrophic neurites (Stephenson et al., 1992; Kalaria et al., 1993; Pluta et al., 1994a; Dietrich et al., 1998; Pluta, 2000a; Chen et al., 2004; van Groen et al., 2005; Pluta et al., 2009). In Alzheimer’s disease, a predominance of neuronal mRNA in amyloid plaques has been observed, suggesting that amyloid plaques develop in areas where neurons die (Ishimaru et al., 1996a; Ginsberg et al., 1999). The presented results support the hypothesis that the amyloid precursor protein gene is overexpressed in neurons damaged and/or with loss of functional innervation, and therefore the early development of diffuse amyloid plaques in Alzheimer’s disease may be a result of neuronal degeneration (Kocki et al., 2015; Pluta et al., 2016a). In single experiments on cerebral ischemia-reperfusion, the presence of senile plaques was observed (van Groen et al., 2005). Some studies confirm that the formation of amyloid plaques and neurofibrillary tangles is a reactive response to ischemic neuronal damage and is not strictly related to the development of dementia (Ishimaru et al., 1996a; Armstrong, 2006). However, amyloid is neurotoxic and may trigger secondary processes in the event of neuronal ischemia. Other studies also indicate that tau protein modifications are a consequence of neurodegenerative mechanisms following ischemia within the neuron body after damage to synaptic connections in the brain neuronal network (Sinigaglia-Coimbra et al., 2002). Finally, tau protein, one of the main hallmarks of Alzheimer’s disease, exacerbates brain parenchyma damage in experimental models of cerebral ischemia by tau protein-mediated iron export and tau protein-dependent excitotoxicity (Bi et al., 2017; Tuo et al., 2017). It can be concluded that amyloid plaques and tau protein modifications arise independently, but once they do, they may cooperate with each other (Duyckaerts, 2004; Perez et al., 2004). If amyloid and tau protein are the result of neurodegeneration, they are most likely a sign of the late stages of Alzheimer’s disease.

We present a hypothetical scheme that fits very well with the ischemic basis of Alzheimer’s disease. In our assumption, Alzheimer’s disease begins to develop when at least two pathological phenomena converge: cerebral ischemia and chronic ischemic failure of the blood-brain barrier. These two events cause acute and chronic neuronal death, ultimately leading to their atrophy and abnormal functioning of the neuronal network, as well as chronic ischemic dysfunction of the blood-brain barrier, which affects the accumulation of amyloid and tau protein in the surrounding brain tissue. It has been noted that the extent and scale of damage to the blood-brain barrier are minimal but permanent, which is of great importance for brain tissue, and the damage appears to accumulate over time (Pluta, 2003; Pluta et al., 2023b). A transgenic model of Alzheimer’s disease in which amyloid accumulates without neuronal loss, e.g., in the hippocampus directly supports this idea (Games et al., 1995; Schmitz et al., 2004; Armstrong, 2006). There is increasing evidence that the neuropathology of Alzheimer’s disease originates from ischemic pathology (Pluta et al., 1994a; Pluta et al., 1996; Pluta, 1997; Etiene et al., 1998; Kalaria, 2000; Pluta, 2001; Pluta, 2004a; Pluta, 2004b; Pluta et al., 2009; Pluta et al., 2013a; Pluta et al., 2013b; Salminen et al., 2017). All indications are that the “amyloid hypothesis” and the “ischemic theory” of Alzheimer’s disease can together fully explain the brain neurodegeneration characteristic of Alzheimer’s disease. Therefore, the accumulation of different amyloid precursor protein epitopes in the post-ischemic brain and the ischemia itself likely constitute a vicious cycle leading to neurodegeneration with dementia (Ishibashi et al., 2006; Kiryk et al., 2011). Progressive neuronal death after an ischemic episode may be caused not only by processes initially activated during ischemia but also by the permeability of the blood-brain barrier to amyloid and tau protein, which contributes to additional changes in ischemic neurons (Pluta et al., 1996; Pluta et al., 1997b; Pluta, 2005; Banks et al., 2017). The basic thesis of our reasoning is that the neuropathology observed in Alzheimer’s disease is a chronic process, starting from primary ischemic neuronal damage (Pluta, 1997; Pluta, 2000a; Pluta, 2002a; Pluta, 2002b; Pluta et al., 2009), to the well-established extravasations of amyloid and tau protein from blood across ischemic blood-brain barrier (Pluta et al., 1996; Pluta et al., 1997b; Pluta et al., 1999; Pluta et al., 2000; Pluta, 2003; Pluta et al., 2003; Pluta, 2005; Pluta et al., 2005; Banks et al., 2017; Pluta et al., 2023b) culminating in the formation of amyloid plaques and neurofibrillary tangles.

## 6 Conclusion

Over the past 3 decades, increasing evidence has accumulated indicating a close link between episodes of cerebral ischemia and Alzheimer’s disease. The relationship between Alzheimer’s disease and cerebrovascular diseases was supported by the following facts: Alzheimer’s disease increases the likelihood of cerebral ischemia and *vice versa*, as well as the fact that amyloid deposits in the brain parenchyma of Alzheimer’s disease patients have a toxic effect on neurons and the vascular system. The accumulated data clearly indicated an association between a history of ischemic brain injury and the development of Alzheimer’s disease. It was presented that, people with a history of cerebral ischemia had a higher risk of developing Alzheimer’s disease and more severe symptoms of dementia. The risk of developing Alzheimer’s disease increases with age, which many experts believe may be the main cause of the disease, and one of these age-related causes is microinfarction. It is known that in the aging brain, many imperceptible, minor ischemic events occur over the years and become more noticeable over time. The cause of microinfarcts is occlusion of microvessles, resulting in a small infarct size in the surrounding tissue.

In this review, we point out the key role of cerebral ischemia in the initiation, progression, and regulation of Alzheimer’s disease-related processes. Cerebral ischemia induces a stereotypical pattern of selective neuronal degeneration that mimics the same phenomenon in Alzheimer’s disease. Furthermore, progressive degeneration of the hippocampus following ischemia was revealed. A transient episode of cerebral ischemia caused delayed death of pyramidal neurons in the CA1 region of the hippocampus, which was associated with the development of chronic neuroinflammation and blood-brain barrier permeability.

Recent evidence indicates that cerebral ischemia simultaneously induces neuronal death and Alzheimer’s disease-related genes. It is likely that Alzheimer’s disease results from a complex interaction between ischemic neuronal damage and the susceptibility of specific Alzheimer’s disease-associated genes to ischemic factor. In this review, we present ischemic induction of genes such as α-, β- and γ-secretase, amyloid precursor protein, apoptosis, autophagy, mitophagy, tau protein, α-synuclein, LRP1, RAGE, and apolipoproteins A1, E and J, which play a key role in the development of Alzheimer’s disease. This article summarizes the latest research findings supporting the hypothesis that genes and their proteins associated with Alzheimer’s disease play a significant role in brain damage caused by ischemia and reperfusion and that an ischemic episode is a necessary and major trigger for the onset and progression of Alzheimer’s disease. Referring to the latest exciting findings, in this review we have combined results from a genomic and proteomic perspective after cerebral ischemia in relation to the development of Alzheimer’s disease. Evidence from experimental and clinical studies has shown that the slow progressive impairment of cognitive function cannot be explained solely by the direct effect of ischemic brain damage, but rather by the progressive influence of additive factors after ischemic damage, e.g., factors related to Alzheimer’s disease. The above suggestions were confirmed by ischemic overexpression of amyloid precursor protein and its metabolizing secretases genes, which may have a strong impact on cognitive decline during the recirculation period, additionally damaging neurons and their networks. Amyloid production increases after cerebral ischemia and negatively affects memory. Moreover, pathological modification of tau protein after ischemia and deposition of α-synuclein may further destroy neurons and impair synaptic function, exacerbating cognitive deficits. Functional changes precede the final degeneration of neuronal cells in sectors selectively susceptible to ischemic episodes.

Several months after ischemia-reperfusion, white matter rarefaction was revealed, and a significant increase in this phenomenon was observed with prolonged survival. In animals that survived for at least 1-year post-ischemia, severe brain atrophy was observed, indicating active, slowly progressive neuropathological processes. The above observations confirm the neuropathological processes we observed, which last much longer than the acute phase. The characteristics of brain neuropathology observed in experimental cerebral ischemia are virtually identical to the degenerative processes in Alzheimer’s disease.

Ischemia-reperfusion-induced brain damage has been shown to cause progressive and irreversible cognitive deficits with an Alzheimer’s disease phenotype, i.e., problems with learning new information in the short-term and memory loss in the long-term post-ischemia survival, suggesting that these dysfunctions result from impaired memory and recall. These types of changes have also been found in Alzheimer’s disease, meaning that cerebral ischemia-reperfusion can be considered a useful experimental model for understanding the processes responsible for causing dementia.

**FIGURE 1:**
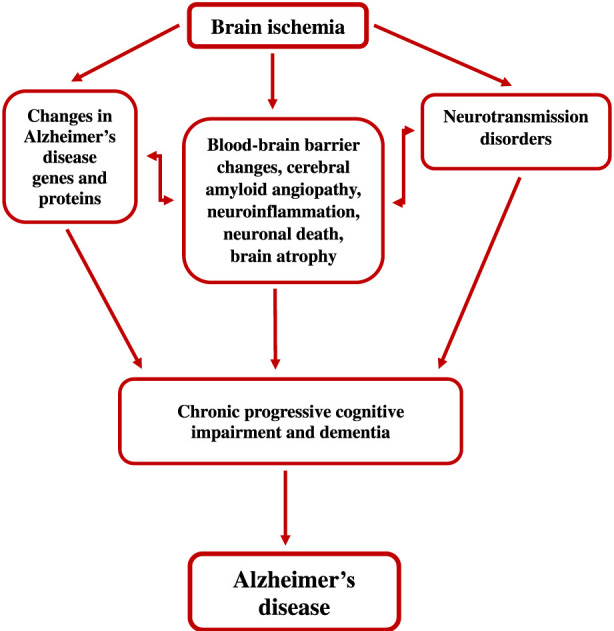
Relationship between brain ischemia and Alzheimer’s disease.

Knowledge of the common molecular mechanisms and processes that contribute to post-ischemic brain neurodegeneration and the development of Alzheimer’s disease provides new opportunities for understanding the ultimate etiology of Alzheimer’s disease. Understanding the processes underlying the association between ischemia-inducing Alzheimer’s disease genes and proteins and the risk of developing Alzheimer’s disease will provide the most anticipated targets for the development of causal treatments for Alzheimer’s disease. Therefore, the overexpression of Alzheimer’s disease-associated genes and the strong staining of different amyloid precursor protein epitopes in the post-ischemic brain, as well as the ischemia itself, likely constitute a self-reinforcing vicious cycle that drives the development of neurodegeneration in Alzheimer’s disease (Figure 1).

Although the precise molecular processes involved in neurodegenerative diseases and neuronal susceptibility to damage remain unknown, reduced expression of the neuronal defense gene α-secretase after brain injury induced by ischemia makes neurons less resistant to damage. The current challenge is to find ways to increase the patient’s adaptive reserves to combat ischemia-related deficits and support neuronal survival. Therefore, it is becoming increasingly important to study the influence of ischemic pathways on neurodegeneration to gain more insights into how to intervene as the disease progresses. In the future, it is likely that manipulation of ischemia itself and the genes activated by ischemia and their proteins will offer new hope for developing causal treatments so urgently needed to prevent or treat neurodegenerative diseases such as Alzheimer’s disease.
